# Cardiac Tamponade: A Rare Manifestation of Familial Mediterranean Fever

**DOI:** 10.1155/2022/8334375

**Published:** 2022-02-07

**Authors:** Abdolreza Malek, Tina Zeraati, Ariane Sadr-Nabavi, Niloofar Vakili, Mohammad Reza Abbaszadegan

**Affiliations:** ^1^Department of Pediatrics, Faculty of Medicine, Mashhad University of Medical Sciences, Mashhad, Iran; ^2^Medical Genetics Research Center, Mashhad University of Medical Sciences, Mashhad, Iran; ^3^Kidney Transplantation Complications Research Center, Mashhad University of Medical Sciences, Mashhad, Iran; ^4^Department of Medical Genetics & Molecular Medicine, Faculty of Medicine, Mashhad University of Medical Sciences, Mashhad, Iran; ^5^Academic Center for Education, Culture, and Research (ACECR)-Khorasan Razavi, Mashhad, Iran; ^6^Institute of Neurogenomics, Helmholtz Zentrum München, Munich, Germany; ^7^Institut für Humangenetik, Technische Universität München, Munich, Germany

## Abstract

Familial Mediterranean fever (FMF) typically presents with recurrent attacks of fever and serosal inflammation with peritoneum, pleura, and synovium. We usually do not expect pericardial involvement at the early stages. FMF is an autoinflammatory disease, usually inherited with an autosomal recessive pattern. The patients typically have biallelic mutations in the MEFV gene, located on chromosome 16. Colchicine is the first-line treatment of FMF, which not only plays a crucial prophylactic role regarding the attack episodes, but also prevents amyloidosis. Colchicine resistance and intolerance in FMF patients have been rarely reported. Alternative anti-inflammatory agents are understood to be helpful in such cases. We describe a 13-year-old boy referred to our pediatric department complaining of chest pain, dyspnea, and tachycardia. Due to the massive pericardial and pleural effusion, a pericardiocentesis was performed, and a chest tube was inserted. Cardiac tamponade was considered as the initial diagnosis. After a month, he faced another episode of pleuritic chest pain, fever, tachycardia, and pleural and pericardial effusion. Evaluation for probable differential diagnoses including infection, malignancy, and collagen vascular disease showed no remarkable results. Finally, the mutation found by whole exome sequencing was confirmed by direct Sanger sequencing revealing a heterozygote c.44G > C (p.Glu148Gln) mutation in exon 2, confirming the clinical diagnosis of familial Mediterranean fever. Since he seemed to be nonresponsive to the maximum standard dose of colchicine, 100 mg of daily dapsone was added to his treatment regimen, which controlled the attack episodes well. FMF, while rarely initiated with cardiac manifestation, should be considered in patients with any early signs and symptoms of cardiovascular involvement.

## 1. Introduction

Familial Mediterranean fever (FMF) is an autoinflammatory disease, typically presented with recurrent fever episodes in addition to serosal inflammation symptoms. However, cardiac involvement mainly occurs in the advanced stages of the disease, primarily due to the growing inflammation and sedimentation of amyloid in the vessels. Basically, the condition is rarely initiated by cardiac symptoms [1].

Colchicine is the treatment of choice for FMF patients; however, there have been reported cases from different parts of the world whose disease was not controlled only by colchicine. Dapsone is considered to be added to colchicine for those not responding to the standard colchicine therapy and those who cannot tolerate higher doses of colchicine [2]. The accurate definition of colchicine resistance in FMF patients has not been well established. Various factors have been considered in the literature, including attack frequency, attack duration, physician assessment, clinical symptoms, and the level of acute phase reactants.

We hereby describe a case of FMF diagnosed based on the clinical symptoms and the findings of direct Sanger sequencing. Severe presentation is not expected in FMF patients with exon 2 mutations; however, our case was presented with pericarditis and pleurisy for the first time. Moreover, the attacks were not controlled prior to the addition of Dapsone to the colchicine regimen.

## 2. Case Presentation

A 13-year-old boy presented to Imam Reza hospital in Mashhad with chest pain and dyspnea. His parents denied any remarkable past medical history but mentioned mild influenza a few days before admission. His parents were not relatives, and his family history included some cases of arthritis and autoimmune diseases among his paternal cousins. A sepsis workup and a cardiopulmonary assessment were initiated. Labs were positive for an elevated sedimentation rate (ESR) and an elevated C-reactive protein, in addition to mild anemia (Table 1). Cardiomegaly was observed in the chest X-ray. A bilateral pleural effusion, dominant in the right side, was found through chest ultrasound. Furthermore, the presence of fluid with a 25 mm diameter around his heart was confirmed. Massive pericardial effusion was considered based on the findings of echocardiography. The electrocardiogram showed alternans cardiac waves; however, cardiac biomarkers were negative. As his signs and symptoms were strongly suggestive for cardiac tamponade, pericardiocentesis was performed, and a chest tube was inserted concerning pleural effusion. Subacute fibrinous pericarditis associated with reactive mesothelial hyperplasia was observed through the histologic assessment of the pericardium. The pericardial fluid analysis and culture were not suggestive of neither malignancy nor infection. Finally, he was discharged and placed on oral clindamycin (600 mg every 6 hours) and cefixime (400 mg once a day).

After a month, he was referred for the second time, complaining of fever, tachycardia, and pleuritic chest pain. Parahilar haziness and peribronchial thickening were seen in his chest X-ray. The chest ultrasound showed left-sided pleural effusion, and it confirmed the presence of fluid around the heart with a 7 mm diameter. His cardiac biomarkers were negative. Laboratory results were significant for leukocytosis 13.34*∗*10^3/uL and an ESR of 57 mm/hr. Blood culture, urine culture, and serologic tests were negative (Table 1). The patient fulfilled major Tel-Hashomer criteria for FMF (recurrent febrile episodes accompanied by pleurisy). Besides, he fulfilled one major and two minor Livneh FMF criteria, including unilateral pleuritis or pericarditis accompanied by severe attacks requiring bed rest; at the same time, there were some attack-free intervals, and his age was below 20 years [3]. Therefore, following whole exome sequencing, direct Sanger sequencing was performed to confirm the mutation and the diagnosis of FMF. The mutation, c.44G > C (p.Glu148Gln), was detected in exon 2 of the MEFV-gene in the heterozygote state, which is typically associated with FMF. Hence, he was placed on 1 mg of daily colchicine, but it did not seem to be effective. After six months, the colchicine dose was increased to 2 mg per day, which resulted in significant symptom improvement during the following six months. However, he faced the side effects of colchicine, such as an increase of liver enzyme levels. Tapering the colchicine dose resulted in the recurrence of disease attacks and an increased rate of weekly hospitalization. The clinical manifestation and essential laboratory data, including WBC, ESR, CRP, and PLT during hospitalization in different episodes and the interval between the episodes, are recorded (Table 2). Apparently, the serum level of acute phase reactants was determined within the normal range whenever the patient had no symptoms. After six months, 100 mg of daily dapsone was added to his treatment regimen. It was so effective that he experienced only one attack in two years.

## 3. Discussion

Familial Mediterranean fever is an autoimmune disease resulting from mutations in the MEFV gene, which encodes pyrin, an inflammasome protein [4]. The gene is located on chromosome 16, and the mutations in this gene are accompanied by both autosomal recessive and autosomal dominant patterns of inheritance. The mutation inherited in an autosomal recessive pattern is usually considered homozygous or compound heterozygous, while the mutation inherited by an autosomal dominant pattern is heterozygous. Most of the identified mutations are located on exon 10, associated with more severe disease, in addition to several mutations on exon 2, which are usually associated with milder symptoms [5]. In 2019, Bilge et al. [6] conducted a study to compare the disease severity of FMF patients with identified mutations located in exon 10 and exon 2. Their findings suggested that amyloidosis, arthritis, and erysipelas-like erythema (ELE) were more frequent in patients with a mutation in exon 10. However, the other symptoms, including peritonitis, fever, pleuritis, vasculitis, myalgia, and chronic renal failure (CRF), were similarly frequent in both groups.

In 2018, Barut et al. [7] conducted a study evaluating the demographic characteristics, clinical manifestations, gene mutations, and responses to the treatment of 708 FMF patients. They reported that the M694V mutation, responsible for the most symptoms, is the most frequent mutation in FMF patients. The gene is expressed in 63.8% of patients who are nonresponsive to colchicine, while only 17% of complete colchicine responders have the mutation.

In 2017, Yoldas et al. [8] from Turkey reported two cases of massive pericardial effusion, later diagnosed with FMF according to clinical and laboratory findings. Their first case was a 13-year-old girl with chest pain, dyspnea, fever, tachycardia, and distant heart sounds. Laboratory findings confirmed a low level of hemoglobin (Hb: 9.7 mg/dl) and an increased level of ESR and CRP without leukocytosis. She was diagnosed with purulent pericarditis due to the accumulation of exudative pericardial fluid; hence, the antibiotic treatment was applied for her. In spite of the appropriate treatment, the amount of pericardial fluid increased massively in four days. The evaluation for connective tissue disorders showed heterozygous R202Q, A165A, G138G, and D102D mutations in exon 2, suggestive for FMF [7]. The second case was a 10-year-old boy referred to the emergency department complaining of chest pain, dyspnea, and cough for two weeks. Muffled heart sounds, tachycardia, and tachypnea were detected through physical examination. Laboratory studies revealed an increased level of acute-phase reactants. Massive pericardial effusion was confirmed by echocardiography, and one liter of hemorrhagic pericardial fluid was drained. She was initially diagnosed with purulent pericarditis and was placed on broad-spectrum antibiotics, but the pericardial fluid culture was negative. Finally, FMF gene analysis revealed homozygous D102D, G138G, A165A, and R202Q mutations in exon 2. Since colchicine treatment failed to reduce the pericardial effusion and stop FMF attack episodes, naproxen was added to his treatment regimen, followed by an acceptable treatment response.

A similarity between the Turkish reported case [8] and our case is that they both manifested cardiac symptoms, in addition to colchicine resistance. As Soriano et al. claimed in their review article, published in 2020, the benefits of colchicine treatment were described in 1972 for the first time. Its efficacy and potential to prevent FMF attack episodes have been proven through numerous clinical trials since then [9]. The total dose of oral colchicine in children with FMF is 0.5 to 2 mg per day, which is usually well-tolerated. Yet, some gastrointestinal adverse events and increased levels of transaminases have been reported following colchicine treatment with the therapeutic dose. Besides, approximately 5% of patients have remained colchicine resistant [10]. For decades, rheumatologists have been establishing fixed criteria to address colchicine resistance identically. They have suggested various measures, including Ben-Chetrit et al. [14], Hentgen et al. [15], and Ozen et al. [16] (10). It seems that the most convincing criteria published to date are those suggested by the EULAR (European League Against Rheumatism). According to EULAR, compliant patients receiving the maximum tolerated dose of colchicine for six months are considered colchicine resistant, providing that they experience one or more attacks monthly. An alternative treatment strategy is required for such patients and those who are suspicious of significant subclinical inflammation leading to secondary amyloidosis [10].

The first alternative treatment for colchicine-resistant patients is NSAID. NSAIDs have appeared effective in some FMF cases, most of whom have manifested with myalgia. The other effective anti-inflammatory agents are glucocorticoids. However, these agents are only used in attack episodes in the form of the intravenous methylprednisolone pulse. Thalidomide is another anti-inflammatory agent. It is used in various inflammatory conditions exclusively for dermatological diseases, such as cutaneous lupus erythematosus. In 2006, Turkish rheumatologists added thalidomide and etanercept to the colchicine regimen. The regimen was applied for the colchicine-resistant FMF patients. Four male patients received thalidomide 100 mg/day, replaced with etanercept for 2 of them, due to thalidomide intolerance, and one female patient received etanercept as the first choice due to the expected side effects of thalidomide in future pregnancy. Their results showed that both thalidomide and etanercept might lower abdominal attacks [14]; however, thalidomide is still not considered as an excellent additional therapy for colchicine resistant patients [10]. The other alternative for colchicine is interferon *α* that can suppress acute inflammation; however, it is less effective than other agents [15].

Another remarkable point is the elevated serum level of specific cytokines such as interleukin-1, interleukin-6, and TNF *α* in the patients. Thus, anti-interleukin 1, anti-interleukin 6, anti-TNF, and Janus kinase inhibitors are expected to be beneficial [16].

Finally, the use of dapsone regarding colchicine resistance is recommended. Dapsone is a sulfonamide known for its both antibacterial and anti-inflammatory features. It is mainly used for neutrophil-dominant dermatologic disorders [17]. It is likely to increase the anti-inflammatory effect when added to colchicine, downregulating neutrophil-mediated inflammatory responses. Inhibiting the production of reactive oxygen species and reducing the effect of eosinophil peroxidase on mast cells are the other anti-inflammatory mechanisms of dapsone [18]. Dapsone is also known for its anti-inflammatory effects regarding a great many inflammatory disorders. For instance, in 2019, Rashidian et al. [19] demonstrated the anti-inflammatory effects of dapsone on acetic acid-induced colitis in rats, probably through NF-kB pathway inhibition. In 2012, Salehzadeh et al. in Iran added 2 mg/kg of dapsone to the standard colchicine-based regimen of 10 FMF patients who were nonresponsive to colchicine; the follow-up duration was six months. The results of their study demonstrated that dapsone appears to control attack episodes of FMF in patients who are nonresponsive to colchicine [2]. Dapsone is also inexpensive and available, and it has fewer side effects than other alternative drug choices. Providing we add it to the standard dose of colchicine in colchicine-resistant patients, the attack rate is expected to decrease.

## 4. Conclusion

Familial Mediterranean fever is not usually manifested with cardiac symptoms for the first time. Cardiac involvement usually occurs at the progressed stages of the disease. Yet, tamponade and pericardial effusion may rarely occur as the first attack episodes of FMF. Early diagnosis and applying the anti-inflammatory treatment in FMF are critical to prevent complications such as amyloidosis. Rheumatologists should be aware of these rare manifestations of FMF, and they ought to seek confirmatory investigations such as whole exome sequencing followed by direct Sanger sequencing confirmation of the mutation before finalizing clinical management to avoid misdiagnosis. Besides, the results of gene assessment can be used for genetic counseling to prevent disease occurrence in the next generation. Furthermore, although colchicine is the treatment of choice in FMF patients, some cases do not respond to the therapeutic doses, and they experience adverse effects with escalating doses. Dapsone seems to be an acceptable alternative which not only reduces the attack episodes but also prevents amyloidosis when it is added to colchicine.

## Figures and Tables

**Table 1:** Laboratory results at first and second admission.

Laboratory tests		First admission	Second admission
Urea		Not determined	25 mg/dl
Cr		Not determined	0.8 mg/dl
Uric acid		Not determined	6.7 mg/dl
Na		Not determined	139–137 mEq/L
K		Not determined	3.5–4 mEq/L
AST		Not determined	18 U/L
ALT		Not determined	22 U/L
LDH		Not determined	376 U/L
CPK		Not determined	37
CK-MB		12	9
TPI		—	—
ESR		11	57
CRP		37.9	Not determined
TSH		6 *μ*U/dl	
T4		0.8 *μ*g/dl	
ASO		Not determined	100
Ferritin		Not determined	317 ng/mL
VBG	pH	Not determined	7.329
	HCO3	Not determined	18.9
	PCO2	Not determined	34.4
	PO2	Not determined	30
	O2SAT	Not determined	53.4

ACE		Not determined	
CBC	WBC	9.2*∗*10^3^ microliter	13.34
	PMN	77.9%	75%
	LYMPH	14.1%	20%
	HB	12.3 g/dl	12.1
	PLT	334*∗*10^3^ microliter	203

Wright		Not determined	—
Coombs wright		Not determined	—
2me		Not determined	—
Widal		Not determined	—
ANA		Not determined	—
EBV IgM		Not determined	—
C4		Not determined	—
C3		Not determined	—
CH50		Not determined	—
UC		Not determined	—
BC		—	—

**Table 2:** Clinical and laboratory findings during episodes and between the episodes.

	Episode 1	Episode 2	Episode 3	Episode 4	Episode 5	Between episodes (without attack)	The latest checkup
Clinical manifestation	Weakness, pleuritic chest pain	Abdominal pain	Abdominal pain	Fever, chest pain	Fever, chest pain	No symptoms	No symptoms
WBC	12.76*∗*10^3^ microliter	15.9*∗*10^3^ microliter	9.19*∗*10^3^ microliter	8.52*∗*10^3^ microliter	8.91*∗*10^3^ microliter	12.6*∗*10^3^ microliter	5*∗*10^3^ microliter
ESR	5	52	7	24	99	3	3
CRP	44.61	83	15.1	223	200	2	Not determined
PLT	209*∗*10^3^ microliter	284*∗*10^3^ microliter	190*∗*10^3^ microliter	197*∗*10^3^ microliter	288*∗*10^3^ microliter	187*∗*10^3^ microliter	193*∗*10^3^ microliter

Cr, creatinine; AST, aspartate transaminase; ALT, alanine transaminase; LDH, lactate dehydrogenase; CPK, creatine kinase; CK-MB, creatine kinase-MB; TPI, troponin; ESR, erythrocyte sedimentation rate; CRP, C-reactive protein; TSH, thyroid-stimulating hormone; ASO, antistreptolysin O; VBG, venous blood gas; ACE, angiotensin-converting enzyme; CBC, complete blood count; WBC, white blood cells; PMN, polymorphonuclear leukocytes; lymph, lymphomononuclear cells; Hb, hemoglobin; PLT, platelet count; 2ME, 2-mercaptoethanol; ANA, antinuclear antibodies; EBV IgM, Epstein–Barr virus antibody; UC, urine culture; BC, blood culture.

## Data Availability

Considering the patient privacy as an ethical concern, access to data is restricted.
